# Coronary-Heart-Disease-Associated Genetic Variant at the *COL4A1/COL4A2* Locus Affects *COL4A1/COL4A2* Expression, Vascular Cell Survival, Atherosclerotic Plaque Stability and Risk of Myocardial Infarction

**DOI:** 10.1371/journal.pgen.1006127

**Published:** 2016-07-07

**Authors:** Wei Yang, Fu Liang Ng, Kenneth Chan, Xiangyuan Pu, Robin N. Poston, Meixia Ren, Weiwei An, Ruoxin Zhang, Jingchun Wu, Shunying Yan, Haiteng Situ, Xinjie He, Yequn Chen, Xuerui Tan, Qingzhong Xiao, Arthur T. Tucker, Mark J. Caulfield, Shu Ye

**Affiliations:** 1 Shantou University Medical College, Shantou, China; 2 William Harvey Research Institute, Queen Mary University of London, London, United Kingdom; 3 Royal Free Hospital, University College London, London, United Kingdom; 4 First Affiliated Hospital, School of Medicine, Zhejiang University, Hangzhou, China; 5 First Affiliated Hospital of Shantou University Medical College, Shantou, China; 6 Department of Cardiovascular Sciences, University of Leicester, Leicester, United Kingdom; 7 NIHR Biomedical Research Centre in Cardiovascular Disease, Leicester, United Kingdom; University College London, UNITED KINGDOM

## Abstract

Genome-wide association studies have revealed an association between coronary heart disease (CHD) and genetic variation on chromosome 13q34, with the lead single nucleotide polymorphism rs4773144 residing in the *COL4A2* gene in this genomic region. We investigated the functional effects of this genetic variant. Analyses of primary cultures of vascular smooth muscle cells (SMCs) and endothelial cells (ECs) from different individuals showed a difference between rs4773144 genotypes in *COL4A2* and *COL4A1* expression levels, being lowest in the G/G genotype, intermediate in A/G and highest in A/A. Chromatin immunoprecipitation followed by allelic imbalance assays of primary cultures of SMCs and ECs that were of the A/G genotype revealed that the G allele had lower transcriptional activity than the A allele. Electrophoretic mobility shift assays and luciferase reporter gene assays showed that a short DNA sequence encompassing the rs4773144 site interacted with a nuclear protein, with lower efficiency for the G allele, and that the G allele sequence had lower activity in driving reporter gene expression. Analyses of cultured SMCs from different individuals demonstrated that cells of the G/G genotype had higher apoptosis rates. Immunohistochemical and histological examinations of *ex vivo* atherosclerotic coronary arteries from different individuals disclosed that atherosclerotic plaques with the G/G genotype had lower collagen IV abundance and thinner fibrous cap, a hallmark of unstable, rupture-prone plaques. A study of a cohort of patients with angiographically documented coronary artery disease showed that patients of the G/G genotype had higher rates of myocardial infarction, a phenotype often caused by plaque rupture. These results indicate that the CHD-related genetic variant at the *COL4A2* locus affects *COL4A2/COL4A1* expression, SMC survival, and atherosclerotic plaque stability, providing a mechanistic explanation for the association between the genetic variant and CHD risk.

## Author Summary

People who carry certain variants in their DNA are genetically predisposed to suffer from coronary heart disease (CHD) caused by abnormal tissue buildup (known as atherosclerosis) and blood clotting in the blood vessels of the heart. One of the DNA variants reported to increase CHD risk is named single nucleotide polymorphism rs4773144. It is still unclear as to why this DNA variant has an effect on CHD risk. In this study, by studying blood vessel cells from many people, we found that the DNA variant affects the production of two collagen genes and vascular cell survival. By examining atherosclerotic tissues from many patients, we discovered that the atherosclerotic tissues of patients who carry the rs4773144 variant are structurally more likely to break down and cause blood clotting which can lead to a heart attack. Furthermore, by studying a group of CHD patients, we noticed that those who carry the rs4773144 variant do have higher rates of heart attack. These findings are useful for understanding why this DNA variant has an impact on CHD risk and suggest that preserving adequate production of these two collagen genes may reduce the risk of heart attack in CHD patients, a potential strategy for development of therapeutics for the disease.

## Introduction

Coronary heart disease (CHD) is a multifactorial disorder caused by both genetic and life-style factors. Genome-wide association studies (GWASs) have revealed a relationship between the disease and genetic variation on chromosome 13q34 [1, 2]. The lead CHD-associated single-nucleotide-polymorphism (SNP) in this genomic region was rs4773144 located in the third intron of the *COL4A2* gene, with the G allele of this SNP associating with increased CHD risk [1, 2]. The molecular and cellular mechanisms underlying this genetic association have, however, remained unclear. Further investigations into such mechanisms are required.

The *COL4A1* and *COL4A2* genes reside next to each other in the head-to-head orientation on chromosome 13q34 and share common transcriptional regulatory sequences [3–9]. These two genes encode the collagen IV protein α1 and α2 chains, respectively [3–9]. Collagen IV is the major constituent of the basement membrane and is essential for its integrity and functionality [10]. In the blood vessel wall, the basement membrane underlies the endothelium and surrounds smooth muscle cells (SMCs) [11]. The basement membrane not only serves as an extracellular scaffold but also regulates cell behavior [10, 12]. Abnormalities of vascular endothelial cells (ECs) and SMCs play important roles in the pathogenesis of atherosclerosis, the vascular pathology underlying CHD [13].

In this study, we sought to investigate the functional effects of the CHD-associated SNP rs4773144 and found that it has an impact on *COL4A2*/*COL4A1* expression, vascular SMC survival, and coronary atherosclerotic plaque stability.

## Results and Discussion

### Effect of SNP rs4773144 genotype on *COL4A2* and *COL4A1* expression

**Fig 1:**
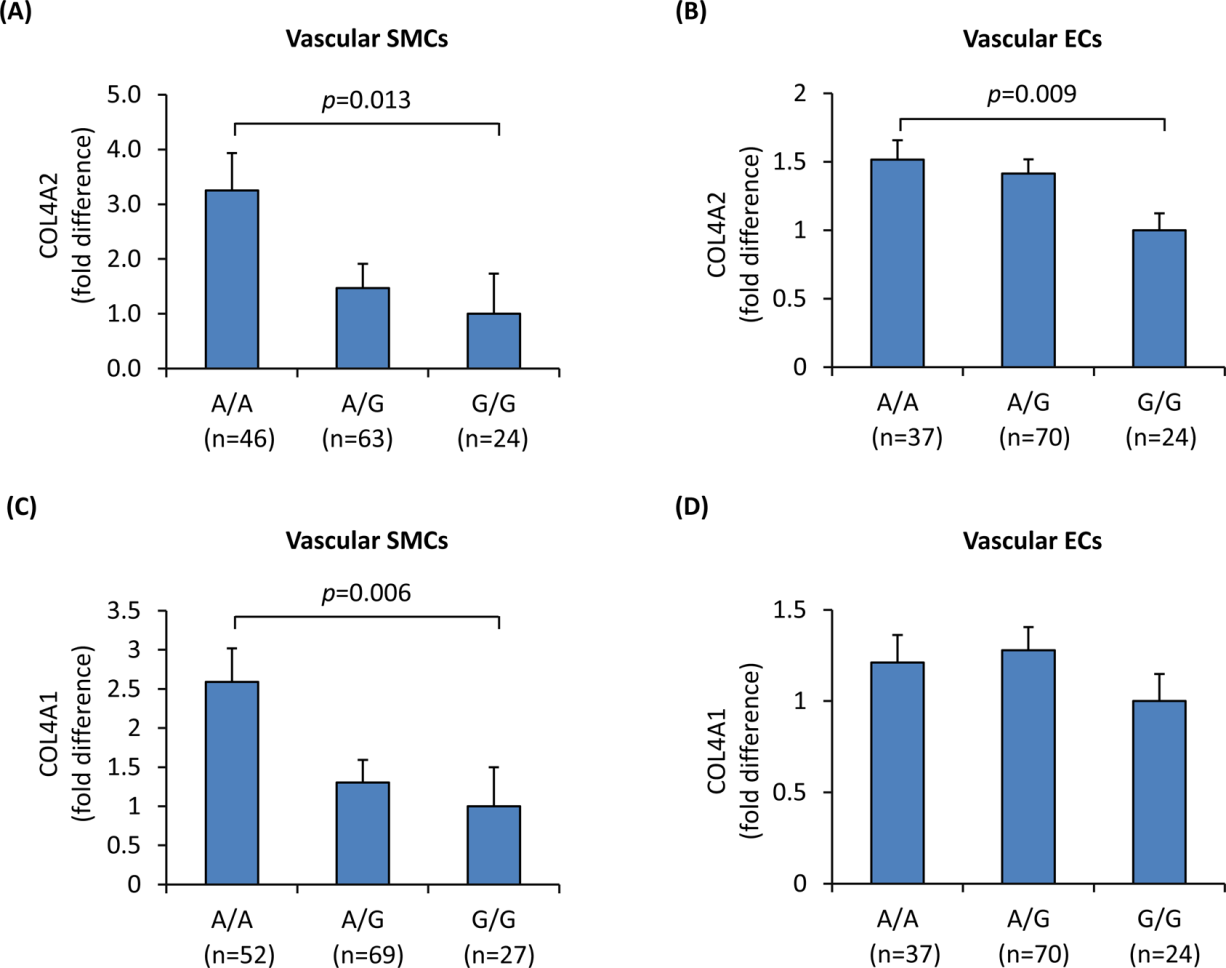
Influence of SNP rs4773144 genotype on *COL4A2* and *COL4A1* expression levels. Primary cultures of vascular SMCs and ECs from different individuals were genotyped for rs4773144 and subjected to quantitative reverse transcriptase–polymerase chain reaction analysis of *COL4A2* and *COL4A1*. Showed in the graphs are relative fold differences in *COL4A2* (**A** and **B**) and *COL4A1* (**C** and **D**) RNA levels in SMCs (**A** and **C**) and ECs (**B** and **D**), respectively. Columns and error bars represent mean and SEM values; *p*-values shown are for an additive genetic model.

To investigate if SNP rs4773144 genotype had an effect on *COL4A2* and *COL4A1* expression, we carried out quantitative RT-PCR assays using total RNA samples extracted from primary cultures of vascular SMCs and ECs from different individuals (n = 148 and n = 137, respectively). The assays showed a genotypic effect of rs4773144 on *COL4A2* RNA levels in both cell types, with the G allele associating with lower expression level in an additive fashion (Fig 1A and 1B), and a similar effect on *COL4A1* RNA levels (Fig 1C and 1D).

The above results suggest that rs4773144 *per se*, or a SNP in linkage disequilibrium with it, influences *COL4A2* and *COL4A1* expression. The *COL4A1* and *COL4A2* genes, situated on chromosome 13q34, are in a head-to-head arrangement, being separated by a bidirectional promoter that drives the transcription of *COL4A1* and *COL4A2* in opposite directions [3–6]. Studies have shown that transcription of these two genes is modulated by regulatory sequences located in the first intron of each gene and the third intron of *COL4A2* [5, 7–9]. SNP rs4773144 resides in the third intron of *COL4A2*, and is in strong linkage disequilibrium (*r*^2^>0.8) with 3 other SNPs (rs4773143, rs7986871 and rs3809346) which are also located in intron 3 of *COL4A2*. A bioinformatics analysis showed that these SNPs reside in a genomic region that has important transcriptional regulatory features including H3k27Ac marks and DNase I hypersensitivity, identified by the ENCODE and Roadmap Epigenomics project (S1 Fig and S2 Fig).

**Fig 2:**
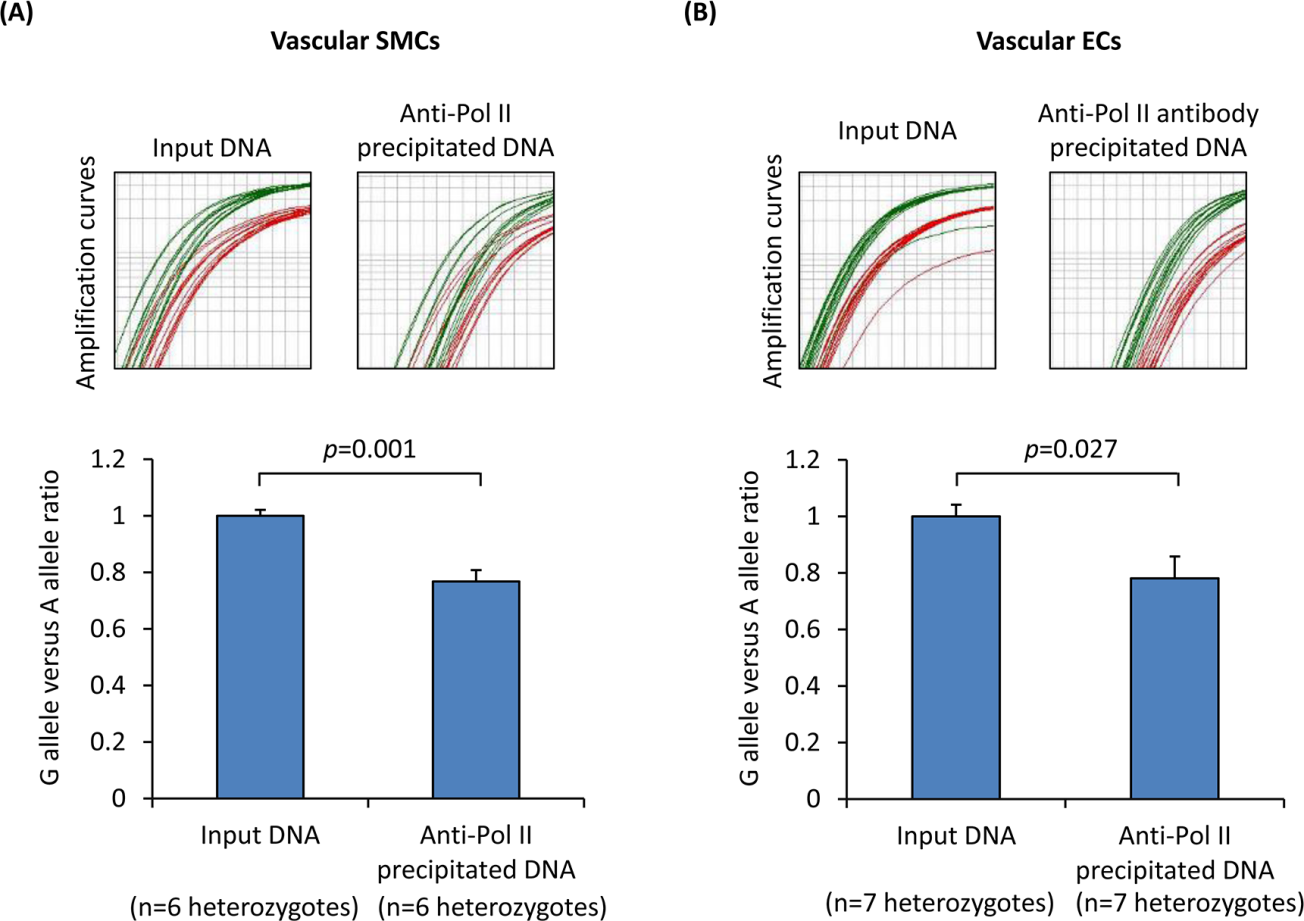
Influence of SNP rs4773144 genotype on gene transcription. Primary cultures of vascular SMCs (A) and ECs (B) of the A/G genotype for SNP rs4773144 from different individuals were subjected to chromatin immunoprecipitation using an antibody against Pol II, followed by an allelic imbalance analysis of SNP rs4773144, with the use of the TaqMan method to determine the ratio of Ct value of the A allele (detected by a VIC fluorescein-labeled probe) versus the Ct value of the G allele (detected by a FAM fluorescent dye-labeled probe). Upper panels show amplification curves of the A allele (red) and G allele (green). Column charts in the lower panels show the relative fold difference in G to A ratio of Ct values between input DNA and anti-Pol II antibody precipitated chromatin DNA. Columns and error bars represent mean and SEM values.

Therefore, we investigated whether SNP rs4773144 affects gene transcription. To this end, we carried out chromatin immunoprecipitation to capture transcriptionally active chromatins using an antibody against RNA polymerase II (Pol II) and then performed allelic imbalance analyses of the immunoprecipitated chromatins from cells that were heterozygous for rs4773144. The analyses showed that in both SMCs and ECs, the ratio of the rs4773144 G allele versus the A allele was lower in chromatin samples precipitated by the anti-Pol II antibody than in non-precipitated chromatin samples (Fig 2A and 2B), indicating that the G allele has lower transcriptional activity than the A allele. An analogous analysis using an anti-H3k27Ac antibody showed a similar trend (S3 Fig).

**Fig 3:**
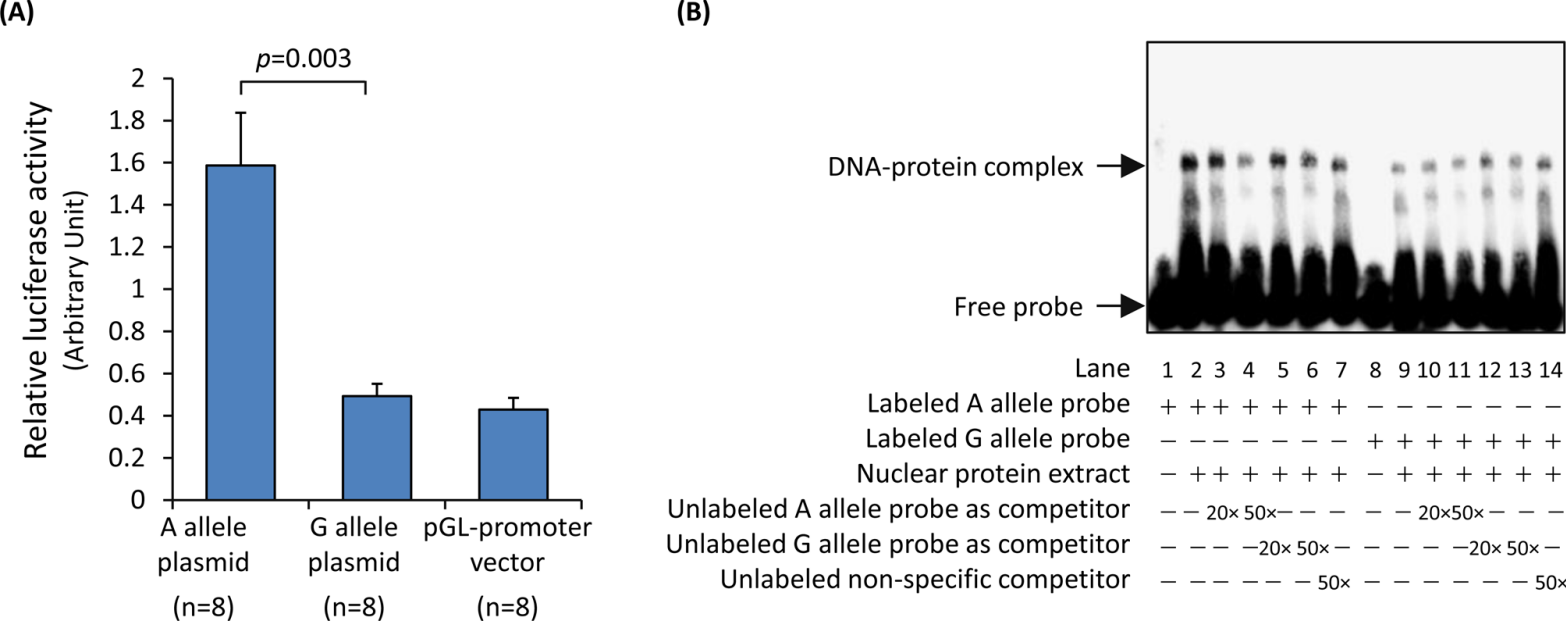
Effect of SNP rs4773144 on transcription modulating activity of a DNA sequence encompassing the SNP and on nuclear protein interaction with the DNA sequence. **(A)** Vascular SMCs were transfected with a firefly luciferase reporter gene plasmid (pGL3-promoter vector) with an insert corresponding to a DNA sequence encompassing the rs4773144 site of the A or G allele, or with the empty plasmid (control), together with a plasmid (pRL-TK) containing a *renilla* luciferase gene to serve as a transfection efficiency reference. At 48 hour after transfection, activities of firefly luciferase and *renilla* luciferase were measured. Columns and error bars represent mean and SEM values of the ratio of firefly luciferase activity to *renilla* luciferase activity. **(B)** A representative image of electrophoretic mobility shift assays. Nuclear protein extracts from vascular ECs were incubated with biotin-labeled probes corresponding to the A allele (lanes 1–7) or the G allele (lanes 8–14) of SNP rs4773144 in the absence or presence of competitors in 20-fold or 50-fold molar excess, as indicated underneath the image.

To further test if the DNA sequence at the rs4773144 site can modulate gene transcription, we performed a luciferase reporter gene assay. In this experiment, SMCs were transfected with a firefly luciferase gene plasmid containing either the rs4773144 G or A allele sequence as well as a *renilla* luciferase gene plasmid to serve as a transfection efficiency reference. The experiment showed that G allele plasmid transfectants had lower firefly luciferase levels than A allele plasmid transfectants (Fig 3A), suggesting that the G allele sequence had lower activity in driving gene transcription.

Further experiments using the electrophoretic mobility shift assay technique showed binding of a nuclear protein to oligonucleotide probes corresponding to the DNA sequence at the rs4773144 site, with lower binding efficiency for the G allele [the DNA-protein complex band had a higher intensity in assays with labeled A allele probe (lane 2) than in assays with labeled G allele probe (lane 9), and was reduced more readily by unlabeled A allele probe (lane 4) than by unlabeled G allele probe (lane 6)](Fig 3B). A bioinformatics analysis showed that the DNA sequence at the rs4773144 site (TTCACGGGA[A/G]) shared similarity with the consensus binding element (TTCNNNNGAA) of the transcription factor STAT3 (S4 Fig). To investigate if the protein in the DNA-protein complex mentioned above was STAT3, we performed electrophoretic mobility super-shift assay using an anti-phospho-STAT3 antibody; however, the formation and mobility of the DNA-protein complex mentioned above was unaffected by the antibody (S5 Fig), suggesting that it may involve a different protein.

### Effect of SNP rs4773144 genotype on cell behavior

**Fig 4:**
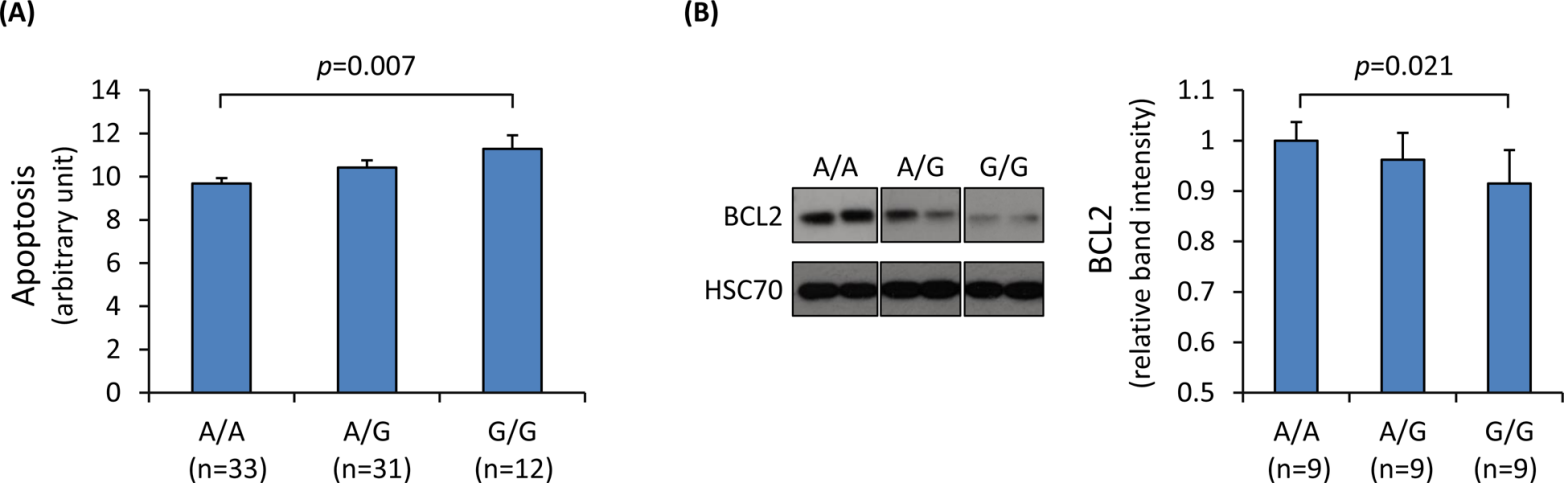
Influence of SNP rs4773144 genotype on apoptosis. **(A)** Primary cultures of SMCs from different individuals were genotyped for rs4773144 and subjected to apoptosis assay. Shown in the graph are relative differences in cell apoptotic rate in different genotype groups. Columns and error bars represent mean and SEM values; *p*-values shown are for an additive genetic model. **(B)** Primary cultures of ECs of different genotypes for rs4773144 were subjected to immunoblot analysis of the anti-apoptotic protein BCL2. Shown on the left are representative immunoblot images and on the right a graphic presentation of BCL2 band intensities standardized against band intensities of the loading control HSC70. Columns and error bars represent mean and SEM values; *p*-values shown are for an additive genetic model.

Collagen IV is the major constituent of the basement membrane and participates in cell-matrix and cell-cell communication [14]. Studies have suggested that collagen IV binds to integrins and activates integrin-mediated intracellular signaling, consequently promoting endothelial cell proliferation and inhibiting apoptosis [15–19]. Integrins promote cell survival and inhibit apoptosis, in part by up-regulating the expression of the anti-apoptotic protein BCL2 [20, 21]. In agreement, we found that knockdown of either *COL4A2* or *COL4A1* in SMCs and ECs resulted in increased apoptosis with a decrease of BCL2 (S6–S9 Figs). Importantly, an analysis of primary cultures of SMCs from different individuals showed an influence of rs4773144 on the rate of apoptosis, with the G allele associating with higher apoptotic rates (Fig 4A). Similarly, there was an association between the G allele and lower levels of the anti-apoptotic protein BCL2 in primary cultures of ECs from different individuals (Fig 4B).

### Effect of SNP rs4773144 genotype on collagen IV levels in atherosclerotic plaques and on plaque stability

**Fig 5:**
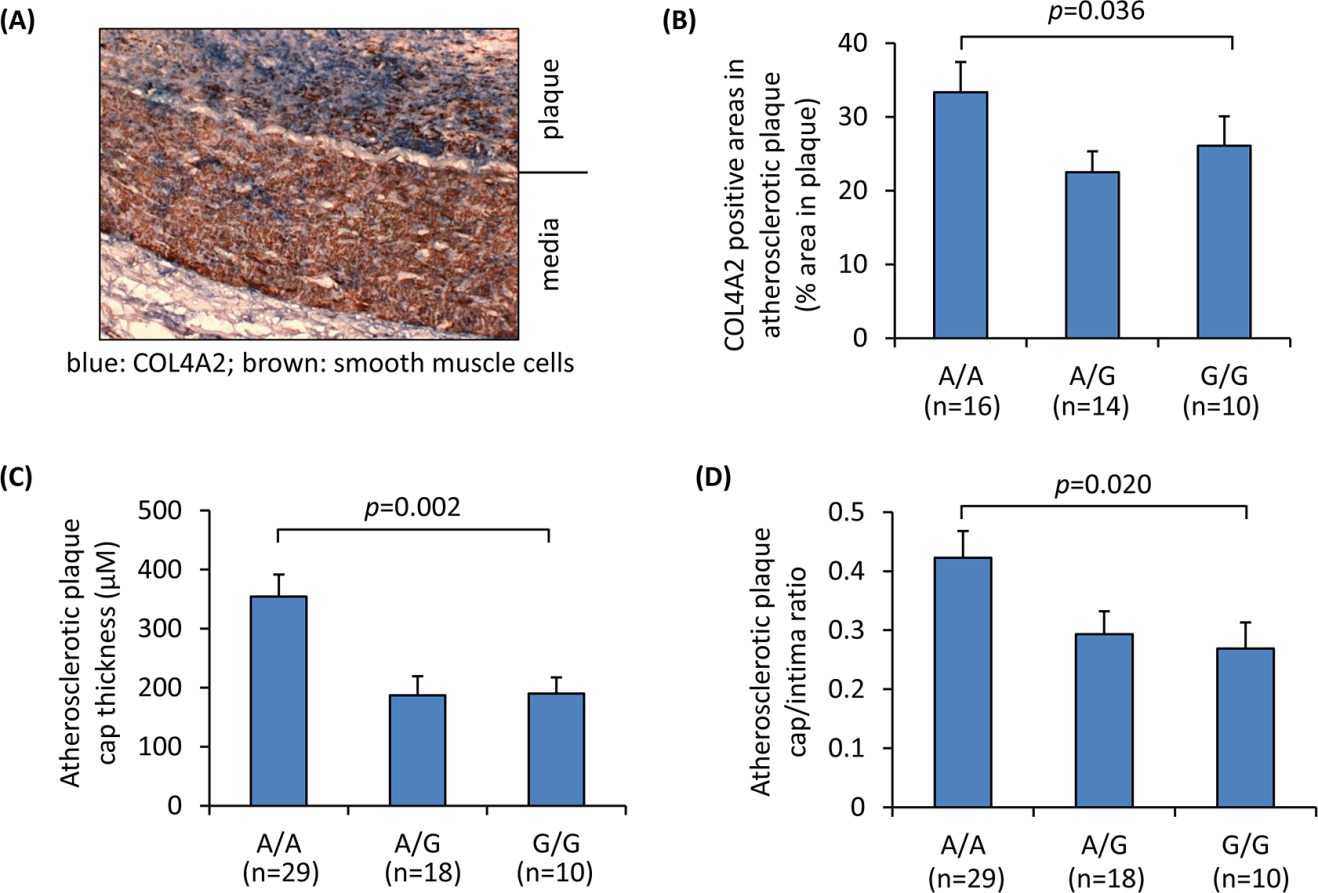
Association of SNP rs4773144 with collagen IV level in atherosclerotic plaques and plaque cap thickness. Atherosclerotic coronary arteries from different individuals were genotyped for SNP rs4773144 and subjected to histopathological analyses using Image-Pro software. **(A)** A representative image of immunostaining, blue color indicates COL4A2 staining, and brown smooth muscle alpha-actin. ×400 original magnification. **(B)** Percentages of COL4A2 immunostain positive areas in atherosclerotic plaques in different genotype groups. **(C)** Atherosclerotic plaque cap thickness in different genotype groups. **(D)** Atherosclerotic plaque cap/intima ratios in different genotype groups. Columns and error bars in **B**-**D** are mean and SEM values; *p*-values shown are for an additive genetic model.

Further to the assays of primary cultures of vascular cells described above, we investigated if there was a relationship between rs4773144 genotype and collagen IV levels in atherosclerotic plaques. In this investigation, atherosclerotic coronary arteries from different individuals were genotyped for rs4773144 and subjected to immunohistochemical analyses using antibodies against COL4A2 and the smooth muscle cell marker SMA (smooth muscle alpha-actin), respectively. The analyses showed a genotypic effect of rs4773144 on the percentages of COL4A2 positive areas in atherosclerotic plaques, with the G allele associating with lower percentages (Fig 5A and 5B).

It is well established that an atherosclerotic plaque typically contains a lipid core covered by a fibrous cap which is primarily composed of SMCs and extracellular matrix proteins [22]. SMC apoptosis can reduce the SMC content and the thickness of the fibrous cap, rendering the plaque prone to rupture, which can trigger thrombosis [23, 24]. Coronary thrombosis is the most common cause of acute coronary ischemic events such as myocardial infarction (MI) [25, 26].

Since our experiments described earlier showed that rs4773144 genotype influenced SMC apoptosis, we investigated whether rs4773144 genotype had an effect on atherosclerotic plaque cap thickness. Histopathological examination of atherosclerotic coronary arteries from individuals of different genotypes for rs4773144 showed a genotypic effect of rs4773144 in plaque cap thickness, with the G allele associating with thinner plaque cap and lower cap/intima ratio (Fig 5C and 5D, S10 Fig and S11 Fig).

Since atherosclerotic plaques with a thin cap are unstable and prone to rupture [23, 24] and coronary atherosclerotic plaque is the primary cause of MI [25, 26], we investigated if there was an association between rs4773144 and MI in CHD patients. We studied a group of patients with angiographically documented coronary disease with >50% luminal stenosis, and observed a genotypic effect of rs4773144, with the G allele associating with higher rates of MI, which remained after adjusting for age, sex, low-density-lipoprotein cholesterol, hypertension, and diabetes mellitus (Table 1).

**Table 1:** Association between SNP rs4773144 and MI incidence/prevalence in CHD patients.

	MI	Non-MI	odds ratio (95% CI), *p-*value	odds ratio* (95% CI), *p-*value
	n (%)	n (%)		
A/A genotype	70 (30.8%)	163 (38.1%)	reference	reference
A/G genotype	106 (46.7%)	206 (48.1%)	1.20 (0.83–1.73), *p* = 0.332	1.20 (0.82–1.76), *p* = 0.356
G/G genotype	51 (22.5%)	59 (13.8%)	2.01 (1.26–3.21), *p* = 0.003	2.09 (1.28–3.41), *p* = 0.003
	frequency	frequency		
A allele	0.54	0.62	reference	
G allele	0.46	0.38	1.39 (1.10–1.75), *p* = 0.005	

*adjusted for age, sex, low-density-lipoprotein cholesterol, hypertension, and diabetes mellitus.

MI, myocardial infarction; CI, confidence interval.

### Summary

There are several novel findings from this study on the effect of the CHD-associated SNP rs4773144 located in the *COL4A2* gene. At the molecular and cellular levels, a genotypic effect on *COL4A2* and *COL4A1* expression in vascular ECs and SMCs and on cell survival was detected. From the examination of *ex vivo* coronary atherosclerotic plaques, we found a relationship between the CHD risk genotype and histological features of plaque instability. Furthermore, in the study of patients with angiographically documented CHD, we observed an association between the risk allele and occurrence of MI. These findings provide a mechanistic explanation for the association between CHD risk and genetic variation at the *COL4A1*/*COL4A2* locus.

Each collagen IV molecule is composed of three α chains forming a triple helical structure, with the classic isoform containing two α1(IV) chains and one α2(IV) chain [27]. The *COL4A1* and *COL4A2* genes, which encode the α1(IV) and α2(IV) chains respectively, reside next to each other on chromosome 13q34 and are transcriptionally co-regulated [3–9]. Severe rare mutations in either *COL4A1* or *COL4A2* have been reported to cause vascular lesions and hemorrhagic stroke in humans [14, 28–31], suggesting that defects of either α1(IV) or α2(IV) can result in similar vascular phenotypes. In agreement, our study shows that SNP rs4773144 genotype affects the expression of both *COL4A1* and *COL4A2*, and the siRNA experiments demonstrate that knockdown of either COL4A1 or COL4A2 in vascular SMCs or ECs induces cell apoptosis (which is line with a reported finding that a frame shift mutation in the *COL4A2* gene increases rates of apoptosis of fibroblasts isolated from an individual carrying the mutation [31]). Apart from providing a mechanistic explanation for the association between rs4773144 and CHD, these results suggest that preserving adequate production of both of these two collagen IV genes can be a potential strategy for developing new therapeutics for the disease.

Data from the ENCODE and Roadmap Epigenomics projects show DNase I hypersensitivity and histone modifications surrounding rs4773144 in a number of different types of cells and tissues including SMCs, ECs, monocytes, T-cells, B-cells, adipose, heart, skeletal muscle, brain, thymus, etc. It is possible that rs4773144 genotype may affect *COL4A2*/*COL4A1* expression not only in vascular SMCs and ECs as demonstrated in this study but also in other cells, and that additional functional mechanisms involving other tissues may also contribute to the association between rs4773144 and CHD.

Over 50 genomic loci have hitherto been identified by GWASs to be associated with CHD risk [32]. However, for many of these loci, the functional mechanisms leading to the genetic effect remain unknown. Functional characterization of these genetic variants can aid the understanding of the underlying biological mechanisms and may facilitate the translation of the genetic discoveries to therapeutic development. The findings of our present study on the CHD-related genetic variant at the *COL4A1*/*COL4A2* locus are pertinent in this context.

## Materials and Methods

### Ethics statement

NRES Committee London–City & East (approval number: 08/H0704/140) and Shantou University Medical College Ethics Committee approved this research.

### Isolation, culture and analyses of primary vascular SMCs and ECs

SMCs were isolated from arteries of umbilical cords from different individuals; ECs were isolated from umbilical cord veins. Isolated SMCs and ECs were subjected to immunocytochemical examinations of the SMC marker SMA, the EC marker von Willebrand factor (vWF), and the fibroblast marker discoidin domain receptor-2 (DDR2), which verified that SMCs were SMA-positive but vWF- and DDR2-negative and that ECs were vWF-positive but SMA- and DDR2-negative. Primary cultures of SMCs and ECs, up to passage 5, were used in experiments of this study.

### Determination of genotypes

Genomic DNA was extracted from cultured SMCs and ECs or from sections of formaldehyde-fixed paraffin-embedded blocks of atherosclerotic coronary arteries using the Wizard SV Genomic DNA Purification System (Promega). rs4773144 genotypes were determined with the use of the TaqMan SNP genotyping assay. Accuracy of the genotyping results was verified by sequencing of a random selection of the samples.

### Quantitative reverse transcriptase–polymerase chain reaction

Total RNA samples were prepared from primary cultures of SMCs and ECs, with the use of the SV Total RNA Isolation System (Promega). RNA was reverse transcribed into cDNA using random primers (Promega) and M-MLV reverse transcriptase (Promega). The resultant cDNA was subjected to real-time polymerase chain reactions for *COL4A1*, *COL4A2*, and *β*-*actin*, respectively, with the use of TaqMan Gene Expression Assays. The 2^-∆∆CT^ method [33] was used to ascertain differences between genotypes in *COL4A1* and *COL4A2* levels standardized against the reference gene *β*-*actin*.

### Chromatin immunoprecipitation and allelic imbalance assays

SMCs and ECs, heterozygous for SNP rs4773144, were crosslinked by incubation in formaldehyde and then incubated with glycine to quench formaldehyde. Subsequently, cells were lysed and chromatin sheared to 200–1,000bp in length by sonication. Aliquots of the samples were incubated with protein G-agarose beads and then further with an anti-human RNA polymerase II antibody (Santa Cruz Biotechnology, sc-9001). DNA-protein-antibody complexes bound to protein G-agarose beads were precipitated by centrifugation and de-crosslinked. Sheared chromatin samples and immunoprecipitated DNA samples were subjected to allelic imbalance analyses of SNP rs4773144, with the use of the TaqMan method to determine the Ct values for the A allele (detected by a VIC fluorescein-labeled probe) and G allele (detected by a FAM fluorescent dye-labeled probe), respectively. Additionally, heterozygous ECs were subjected to chromatin immunoprecipitation using an anti-human H3k27Ac antibody (Abcam ab4729) and then TaqMan assay to determine the G allele versus A allele ratio.

### Luciferase reporter gene assays

A 350 base pair DNA sequence encompassing the rs4773144 site of the A and G alleles, respectively, was amplified by PCR and inserted into the pGL3-promoter vector (Promega) containing a firefly luciferase reporter gene. The resultant construct containing the inserted DNA sequence corresponding to either the A or G allele of rs4773144 was mixed with a plasmid (pRL-TK, Promega) containing a *renilla* luciferase gene and transfected into cultured vascular SMCs. At 48 hours after transfection, the transfectants were lysed, and the activities of firefly luciferase and *renilla* luciferase in the lysates were measured. The ratio of firefly luciferase activity to *renilla* luciferase activity was used as a measurement of the transcription modulating activity of the inserted DNA sequence encompassing the rs4773144 site of the A and G alleles, respectively.

### Electrophoretic mobility shift assays

Biotin-labelled, double-stranded 25-mer oligonucleotides corresponding to the sequences at and surrounding the SNP rs4773144 site were used as probes. The probe sequences were: 5'-CCTTTCACGGGAACTGGGAACTTAA-3' (A allele) and 5’-CCTTTCACGGGAGCTGGGAACTTAA-3’ (G allele), respectively. The probes were individually incubated with nuclear extracts of cultured ECs, in the presence or absence of unlabelled oligonucleotide competitors in molar excess or an anti-phospho-STAT3 antibody (Cell Signaling Technology, #9131), followed by non-denaturing polyacrylamide gel electrophoresis. Free probes and probe-protein complexes were detected using a LightShift Chemiluminescent EMSA kit (Pierce Biotechnology, 20148). Three independent experiments were carried out.

### *COL4A2* or *COL4A1* siRNA knockdown

SMCs and ECs were transfected with either *COL4A2* siRNA (ThermoFisher Scientific, 4457308), *COL4A1* siRNA (ThermoFisher Scientific, AM16708), or control siRNA (ThermoFisher Scientific, 4390843), with the use of Lipofectamine RNAiMAX transfection reagent (Invitrogen, 13778150). COL4A2 or COL4A1 knockdown was verified by immunoblotting analysis.

### Immunoblotting analyses

Cell lysates were prepared by incubating cells with a lysis buffer containing a protease inhibitor cocktail. An aliquot of 20μg proteins from each sample was subjected to Tris-glycine, sodium-dodecyl-sulfate, polyacrylamide gel electrophoresis, followed by standard immunoblotting analysis with an anti-COL4A2 antibody (Abcam, ab69782), an anti-COL4A1 antibody (Abnova, PAB17326), an anti-BCL2 antibody (Abcam, ab32124), an anti-β-actin antibody, or an anti-HSC70 antibody (Santa Cruz, sc7298).

### Cell accumulation and apoptosis assays

SMCs (same number) or ECs (same number) transfected with either the *COL4A2* siRNA, *COL4A1* siRNA or control siRNA, or untransfected primary SMCs (same number) from different individuals, were cultured for 72 hours, and then detached and counted. Additionally, cells were subjected to apoptosis assays with the use of Annexin V-FITC apoptosis detection kit (Beyotime Institute of Biotechnology, C1062, for transfected cells) or Cell Death Detection ELISAPLUS kit (Roche, 11774425001, for untransfected cells) to quantify histone-complexed DNA fragments.

### Immunohistochemical analysis of *ex vivo* coronary artery atherosclerotic plaques

Formaldehyde-fixed paraffin-embedded sections of atherosclerotic coronary arteries from autopsies were deparaffinised, rehydrated, and incubated in sodium citrate for antigen retrieval. The sections were subjected to peroxidase immunostaining with a mouse anti-human smooth muscle α-actin (SMA) antibody (Dako, M-0635). A subset of the collection was subjected to peroxidase/alkaline phosphatase double immunostaining with the mouse anti-human smooth muscle α-actin (SMA) antibody (Dako, M-0635) and a rabbit anti-human COL4A2 antibody (Abcam, ab69782). Chromagens were diaminobenzidine and nitrobluetetrazolium/bromo-chloro-indolyl phosphate, respectively. Images of the sections were captured using a microscope with an imaging system and analyzed using Image-Pro software to determine the sizes of positive immunostain areas, atherosclerotic plaque cap thickness, and intima thickness.

### Patients with angiographically documented coronary artery disease

We studied 1125 consecutive patients undergoing diagnostic or interventional coronary angiography in the First Affiliated Hospital of Shantou University Medical College. All subjects were Chinese. We collected demographic and clinical data including age, sex, plasma levels of total cholesterol, low-density-lipoprotein cholesterol, high-density lipoprotein cholesterol and triglycerides, coronary angiographic findings, incident or prevalent MI diagnosed according to the World Health Organization criteria, systolic and diastolic blood pressure, and the presence or absence of diabetes mellitus. Of the 1125 subjects, 655 had significant angiographically documented CHD as having >50% diameter stenosis in ≥1 major epicardial coronary artery. Among them, a total of 227 subjects had incident or prevalent MI. The study was approved by the appropriate research ethics committee. The data were analyzed anonymously.

### Statistical analyses

Variables not in normal distribution were normalized by logarithmic transformation. Linear regression analyses were performed to test differences between genotypes in *COL4A1* and *COL4A2* expression levels, apoptosis assay result, BCL2 immunoblotting band intensity (standardized against HSC70 band intensity), percentage of COL4A2 stain areas in total atherosclerotic plaque area, atherosclerotic plaque cap thickness, and atherosclerotic plaque cap/intima ratio, in an additive genetic model. Student’s *t*-tests were used to ascertain allelic differences in the allelic expression imbalance assays and the chromatin immunoprecipitation assays. Student’s *t*-tests were also used to test differences between cells transfected with either the rs4773144 A allele plasmid or G allele plasmid in firefly luciferase activity after standardized against *renilla* luciferase activity in the luciferase assays, and between cells transfected with *COL4A2* siRNA or *COL4A1* siRNA and cells transfected with control siRNA in cell count, proliferation assay result, apoptosis assay result, and BCL2 immunoblotting band intensity (standardized against β-actin band intensity), respectively. Logistic regression analyses and chi-squared tests were carried out to ascertain genotypic and allelic association with MI in CHD patients. All *p* values were two-sided.

## Supporting Information

S1 Fig Results of bioinformatics analysis of SNP rs4773144: Data from the ENCODE project. A bioinformatics analysis with the use of the UCSC Genome Browser showed that SNP rs4773144, and 3 other SNPs (rs4773143, rs7986871 and rs3809346) in strong linkage disequilibrium (*r*^2^>0.8) with it, are located in a genomic region that has important transcriptional regulatory features including DNase I hypersensitivity and H3k27Ac marks, identified by the ENCODE project.
(PDF)

S2 Fig Results of bioinformatics analysis of SNP rs4773144: Data from the Roadmap Epigenomics Project. Data from the Roadmap Epigenomics Project show that SNP rs4773144 (position indicated by vertical yellow line) and 3 other SNPs (rs4773143, rs7986871 and rs3809346) in strong linkage disequilibrium (*r*^2^>0.8) with it, are located in a genomic region that has important transcriptional regulatory features including DNase I hypersensitivity and H3k27Ac marks in a large number of cell lines and tissues of different types.
(PDF)

S3 Fig Results of chromatin immunoprecipitation analysis with anti-H3K27Ac antibody. Primary cultures of vascular ECs of the A/G genotype for SNP rs4773144 were subjected to chromatin immunoprecipitation using an antibody against H3K27Ac, followed by an allelic imbalance analysis of SNP rs4773144 by TaqMan assay. Data shown are mean (SEM) values of G allele to A allele ratio in input DNA and anti-H3K27Ac antibody precipitated chromatin DNA.
(PDF)

S4 Fig Results of bioinformatics analysis of SNP rs4773144: Data from RegulomeDB. Data from RegulomeDB show that SNP rs4773144 (position indicated by vertical red line) and 3 other SNPs (rs4773143, rs7986871 and rs3809346) in strong linkage disequilibrium (*r*^2^>0.8) with it, are located in a genomic region with binding of the RNA polymerase II POLR2A subunit and transcription factors, detected by the ENCODE project, and that the DNA sequence at the rs4773144 site shares similarity with the consensus binding element of the transcription factor STAT3.
(PDF)

S5 Fig Formation and mobility of the DNA-protein complex unaffected by anti-STAT3 antibody. A representative image of electrophoretic mobility shift assay. Nuclear protein extracts were prepared from vascular ECs that had been stimulated with interleukin-6 (200ng/mL) and interleukin-6 soluble receptor (200ng/mL) for 20 hours. Nuclear protein extracts were incubated with a biotin-labeled probe corresponding to the A allele of SNP rs4773144 in the absence or presence of an anti-phospho-STAT3 antibody (Cell Signaling Technology, Cat. No. #9131) or competitors in 50-fold molar excess, as indicated underneath the image.
(PDF)

S6 Fig Effect of COL4A2 knockdown on cultured vascular SMCs. Human aortic artery SMCs were transfected with *COL4A2* siRNA or control siRNA. **(A)** Transfected cells were subjected to immunoblotting analysis of the target protein COL4A2 and the housekeeping protein β-actin. Shown in figure are representative immunoblotting images. **(B)** Transfected cells (same number) were cultured on collagen IV coated or uncoated multi-well plates for 72 hours, and then detached and counted. Column chart shows mean and SEM values of cell counts from four independent experiments. **(C)** Transfected cells were subjected to immunoblotting analysis of the anti-apoptotic protein BCL2 and the housekeeping protein β-actin. Upper panel shows representative immunoblotting images. Column chart shows mean and SEM values of BCL2 band intensity standardized against β-actin band intensity from three independent experiments. **(D)** Transfected cells were cultured on collagen IV coated or uncoated multi-well plates, and subjected to apoptosis assays. Images show representative staining results: nuclei of apoptotic cells are in green, and those in non-apoptotic cells are in blue. Column chart shows mean and SEM values of apoptotic cells from four independent experiments.
(PDF)

S7 Fig Effect of COL4A2 knockdown on cultured vascular ECs. Cultured ECs were transfected with *COL4A2* siRNA or control siRNA. **(A)** Transfected cells were subjected to immunoblotting analysis of the target protein COL4A2 and the housekeeping protein β-actin. Shown in figure are representative immunoblotting images. **(B)** Transfected cells (same number) were cultured on collagen IV coated or uncoated multi-well plates for 72 hours, and then detached and counted. Column chart shows mean and SEM values of cell counts from four independent experiments. **(C)** Transfected cells were subjected to immunoblotting analysis of the anti-apoptotic protein BCL2 and the housekeeping protein β-actin. Upper panel shows representative immunoblotting images. Column chart shows mean and SEM values of BCL2 band intensity standardized against β-actin band intensity from three independent experiments. **(D)** Transfected cells were cultured on collagen IV coated or uncoated multi-well plates, and subjected to apoptosis assays. Images show representative staining results: nuclei of apoptotic cells are in green, and those in non-apoptotic cells are in blue. Column chart shows mean and SEM values of apoptotic cells from four independent experiments.
(PDF)

S8 Fig Effect of COL4A1 knockdown on cultured vascular SMCs. Human aortic artery SMCs were transfected with *COL4A1* siRNA or control siRNA. **(A)** Transfected cells were subjected to immunoblotting analysis of the target protein COL4A1 and the housekeeping protein β-actin. Shown in figure are representative immunoblotting images. **(B)** Transfected cells (same number) were cultured on collagen IV coated or uncoated multi-well plates for 72 hours, and then detached and counted. Column chart shows mean and SEM values of cell counts from four independent experiments. **(C)** Transfected cells were subjected to immunoblotting analysis of the anti-apoptotic protein BCL2 and the housekeeping protein β-actin. Upper panel shows representative immunoblotting images. Column chart shows mean and SEM values of BCL2 band intensity standardized against β-actin band intensity from three independent experiments. **(D)** Transfected cells were cultured on collagen IV coated or uncoated multi-well plates, and subjected to apoptosis assays. Images show representative staining results: nuclei of apoptotic cells are in green, and those in non-apoptotic cells are in blue. Column chart shows mean and SEM values of apoptotic cells from four independent experiments.
(PDF)

S9 Fig Effect of COL4A1 knockdown on cultured vascular ECs. Cultured ECs were transfected with *COL4A1* siRNA or control siRNA. **(A)** Transfected cells were subjected to immunoblotting analysis of the target protein COL4A1 and the housekeeping protein β-actin. Shown in figure are representative immunoblotting images. **(B)** Transfected cells (same number) were cultured on collagen IV coated or uncoated multi-well plates for 72 hours, and then detached and counted. Column chart shows mean and SEM values of cell counts from four independent experiments. **(C)** Transfected cells were subjected to immunoblotting analysis of the anti-apoptotic protein BCL2 and the housekeeping protein β-actin. Upper panel shows representative immunoblotting images. Column chart shows mean and SEM values of BCL2 band intensity standardized against β-actin band intensity from three independent experiments. **(D)** Transfected cells were cultured on collagen IV coated or uncoated multi-well plates, and subjected to apoptosis assays. Images show representative staining results: nuclei of apoptotic cells are in green, and those in non-apoptotic cells are in blue. Column chart shows mean and SEM values of apoptotic cells from four independent experiments.
(PDF)

S10 Fig Association of SNP rs4773144 with plaque cap thickness. Atherosclerotic coronary arteries from different individuals were genotyped for SNP rs4773144 and subjected to histopathological analysis. Atherosclerotic plaque cap thickness was analyzed using Image-Pro software. **(A)** Data from sample set one; **(B)** Data from sample set two; **(C)** Results from the two sample sets combined. Data shown are mean and SEM values in different genotype groups; *p*-values shown are for an additive genetic model.
(PDF)

S11 Fig Association of SNP rs4773144 with plaque cap/intima thickness ratio. Atherosclerotic coronary arteries from different individuals were genotyped for SNP rs4773144 and subjected to histopathological analysis. Atherosclerotic plaque cap thickness and whole intima thickness were analyzed using Image-Pro software. **(A)** Data from sample set one. **(B)** Data from sample set two. **(C)** Results from the two sample sets combined. Data shown are mean and SEM values in different genotype groups; *p*-values shown are for an additive genetic model.
(PDF)
