# Supplementary material for: Coronary-Heart-Disease-Associated Genetic Variant at the COL4A1/COL4A2 Locus Affects COL4A1/COL4A2 Expression, Vascular Cell Survival, Atherosclerotic Plaque Stability and Risk of Myocardial Infarction
Source: PLoS Genet. 2016 Jul 7;12(7):e1006127. doi: 10.1371/journal.pgen.1006127 (PMC4936713; doi:10.1371/journal.pgen.1006127)
Supplement: S1 Fig — A bioinformatics analysis with the use of the UCSC Genome Browser showed that SNP rs4773144, and 3 other SNPs (rs4773143, rs7986871 and rs3809346) in strong linkage disequilibrium (r2>0.8) with it, are located in a genomic region that has important transcriptional regulatory features including DNase I hypersensitivity and H3k27Ac marks, identified by the ENCODE project. (PDF) [file pgen.1006127.s001.pdf]

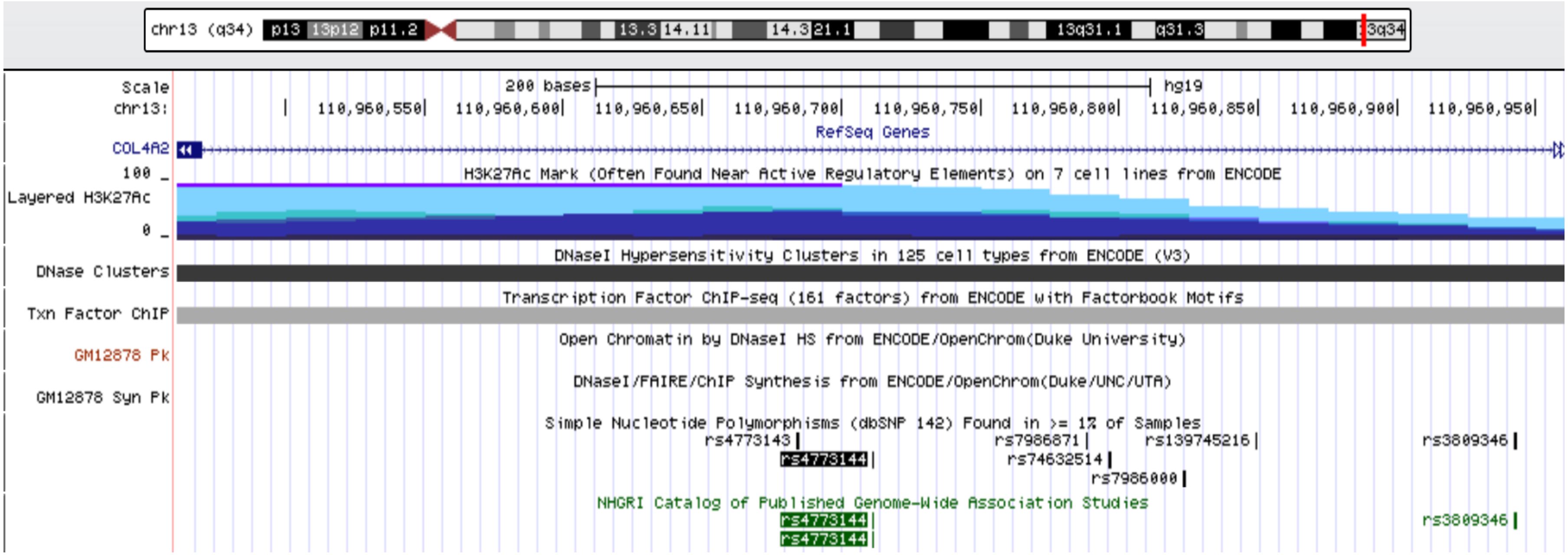

S1 Fig. Results of Bioinformatics Analysis of SNP rs4773144: Data from the ENCODE Project.
A bioinformatics analysis with the use of the UCSC Genome Browser showed that SNP rs4773144, and 3 other SNPs (rs4773143, rs7986871 and rs3809346) in strong linkage disequilibrium (r^2>0.8) with it, are located in a genomic region that has important transcriptional regulatory features including DNase I hypersensitivity and H3k27Ac marks, identified by the ENCODE project.
