# Supplementary material for: Coronary-Heart-Disease-Associated Genetic Variant at the COL4A1/COL4A2 Locus Affects COL4A1/COL4A2 Expression, Vascular Cell Survival, Atherosclerotic Plaque Stability and Risk of Myocardial Infarction
Source: PLoS Genet. 2016 Jul 7;12(7):e1006127. doi: 10.1371/journal.pgen.1006127 (PMC4936713; doi:10.1371/journal.pgen.1006127)
Supplement: S3 Fig — Primary cultures of vascular ECs of the A/G genotype for SNP rs4773144 were subjected to chromatin immunoprecipitation using an antibody against H3K27Ac, followed by an allelic imbalance analysis of SNP rs4773144 by TaqMan assay. Data shown are mean (SEM) values of G allele to A allele ratio in input DNA and anti-H3K27Ac antibody precipitated chromatin DNA. (PDF) [file pgen.1006127.s003.pdf]

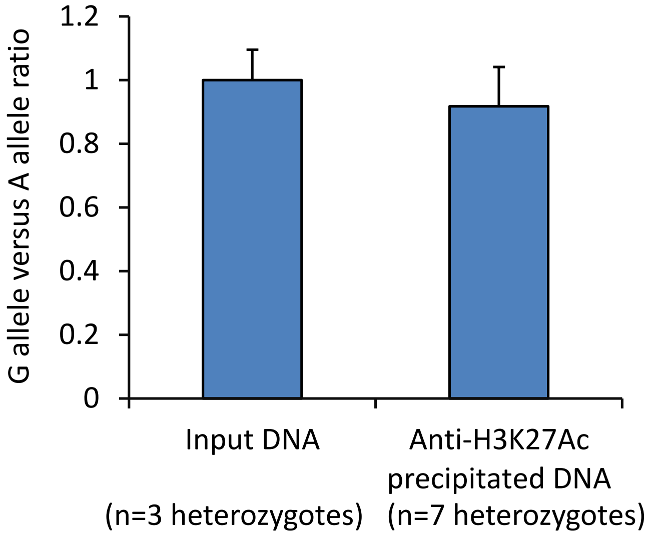

S3 Fig. Results of Chromatin Immunoprecipitation Analysis with an Anti-H3K27Ac Antibody. Primary cultures of vascular ECs of the A/G genotype for SNP rs4773144 were subjected to chromatin immunoprecipitation using an antibody against H3K27Ac, followed by an allelic imbalance analysis of SNP rs4773144 by TaqMan assay. Data shown are mean (SEM) values of G allele to A allele ratio in input DNA and anti-H3K27Ac antibody precipitated chromatin DNA.
