# Supplementary material for: Coronary-Heart-Disease-Associated Genetic Variant at the COL4A1/COL4A2 Locus Affects COL4A1/COL4A2 Expression, Vascular Cell Survival, Atherosclerotic Plaque Stability and Risk of Myocardial Infarction
Source: PLoS Genet. 2016 Jul 7;12(7):e1006127. doi: 10.1371/journal.pgen.1006127 (PMC4936713; doi:10.1371/journal.pgen.1006127)
Supplement: S4 Fig — Data from RegulomeDB show that SNP rs4773144 (position indicated by vertical red line) and 3 other SNPs (rs4773143, rs7986871 and rs3809346) in strong linkage disequilibrium (r2>0.8) with it, are located in a genomic region with binding of the RNA polymerase II POLR2A subunit and transcription factors, detected by the ENCODE project, and that the DNA sequence at the rs4773144 site shares similarity with the consensus binding element of the transcription factor STAT3. (PDF) [file pgen.1006127.s004.pdf]

RegulomeDB Download

Data supporting chr13:110960711 (rs4773144)

Score: 2b

Likely to affect binding

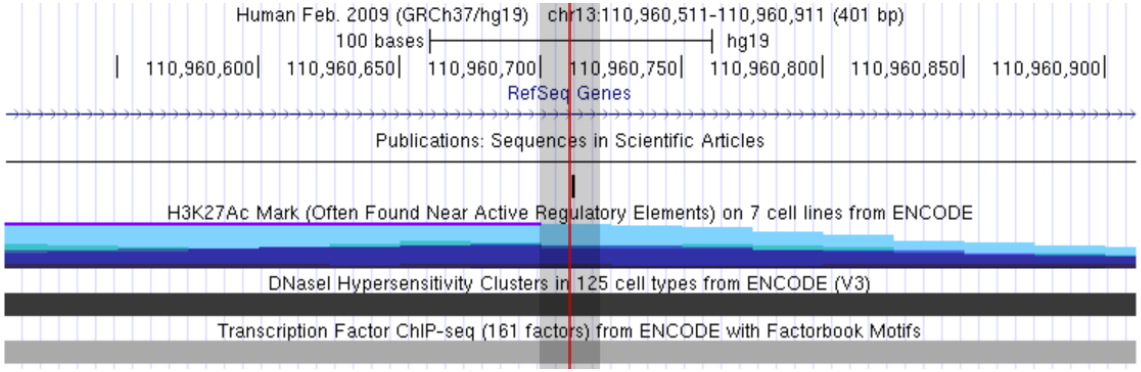

Protein Binding Filter:

Method	Location	Bound Protein	Cell Type	Additional Info	Reference
ChIP-seq	chr13:110960617..110961033	POLR2A	HUVEC		ENCODE
ChIP-seq	chr13:110960607..110961147	POLR2A	MCF10A-Er-Src	01pct	ENCODE
ChIP-seq	chr13:110960657..110961027	POLR2A	ProgFib		ENCODE
ChIP-seq	chr13:110959966..110960876	EZH2	H1-hESC		ENCODE

Motifs Filter:

Method	Location	Motif	Cell Type	PWM	Reference
Footprinting	chr13:110960702..110960716	STAT3:STAT3	Gliobla	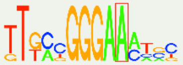	21106904
Footprinting	chr13:110960702..110960716	STAT3:STAT3	H1hesc	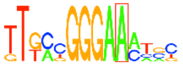	21106904
PWM	chr13:110960702..110960716	STAT3:STAT3		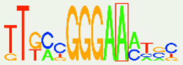	16381825

S4 Fig. Results of Bioinformatics Analysis of SNP rs4773144: Data from RegulomeDB. Data from RegulomeDB show that SNP rs4773144 (position indicated by vertical red line) and 3 other SNPs (rs4773143, rs7986871 and rs3809346) in strong linkage disequilibrium (r^2>0.8) with it, are located in a genomic region with binding of the RNA polymerase II POLR2A subunit and transcription factors, detected by the ENCODE project, and that the DNA sequence at the rs4773144 site shares similarity with the consensus binding element of the transcription factor STAT3.
