# Supplementary material for: Coronary-Heart-Disease-Associated Genetic Variant at the COL4A1/COL4A2 Locus Affects COL4A1/COL4A2 Expression, Vascular Cell Survival, Atherosclerotic Plaque Stability and Risk of Myocardial Infarction
Source: PLoS Genet. 2016 Jul 7;12(7):e1006127. doi: 10.1371/journal.pgen.1006127 (PMC4936713; doi:10.1371/journal.pgen.1006127)
Supplement: S5 Fig — A representative image of electrophoretic mobility shift assay. Nuclear protein extracts were prepared from vascular ECs that had been stimulated with interleukin-6 (200ng/mL) and interleukin-6 soluble receptor (200ng/mL) for 20 hours. Nuclear protein extracts were incubated with a biotin-labeled probe corresponding to the A allele of SNP rs4773144 in the absence or presence of an anti-phospho-STAT3 antibody (Cell Signaling Technology, Cat. No. #9131) or competitors in 50-fold molar excess, as indicated underneath the image. (PDF) [file pgen.1006127.s005.pdf]

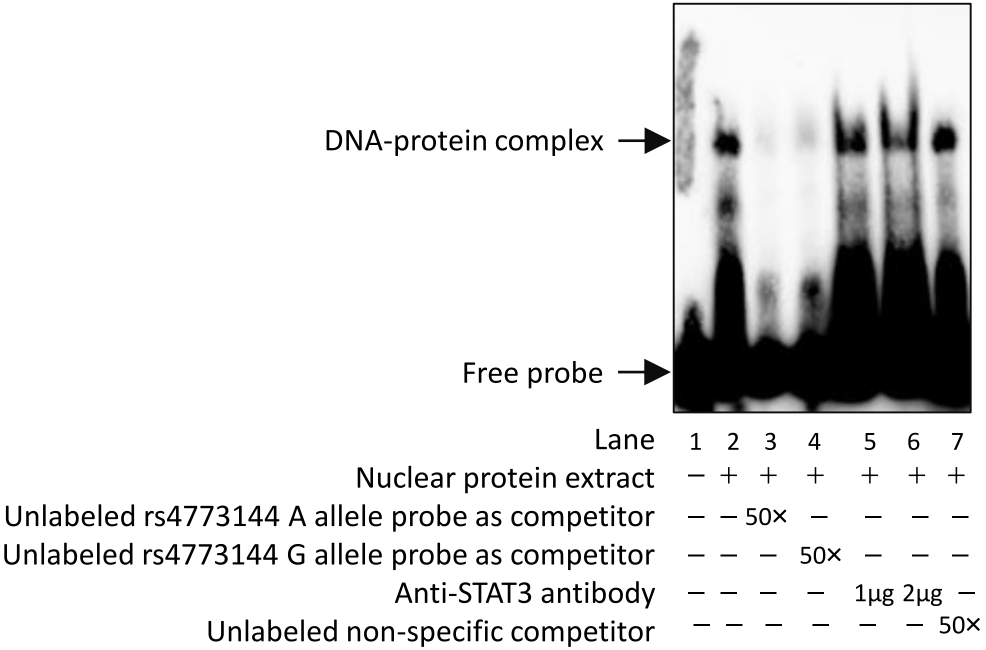

S5 Fig. Formation and Mobility of the DNA-Protein Complex Unaffected by an Anti-STAT3 Antibody. A representative image of electrophoretic mobility shift assay. Nuclear protein extracts were prepared from vascular ECs that had been stimulated with interleukin-6 (200ng/mL) and interleukin-6 soluble receptor (200ng/mL) for 20 hours. Nuclear protein extracts were incubated with a biotin-labeled probe corresponding to the A allele of SNP rs4773144 in the absence or presence of an anti-phospho-STAT3 antibody (Cell Signaling Technology, Cat. No. #9131) or competitors in 50-fold molar excess, as indicated underneath the image.
