# Supplementary material for: Coronary-Heart-Disease-Associated Genetic Variant at the COL4A1/COL4A2 Locus Affects COL4A1/COL4A2 Expression, Vascular Cell Survival, Atherosclerotic Plaque Stability and Risk of Myocardial Infarction
Source: PLoS Genet. 2016 Jul 7;12(7):e1006127. doi: 10.1371/journal.pgen.1006127 (PMC4936713; doi:10.1371/journal.pgen.1006127)
Supplement: S6 Fig — Human aortic artery SMCs were transfected with COL4A2 siRNA or control siRNA. (A) Transfected cells were subjected to immunoblotting analysis of the target protein COL4A2 and the housekeeping protein β-actin. Shown in figure are representative immunoblotting images. (B) Transfected cells (same number) were cultured on collagen IV coated or uncoated multi-well plates for 72 hours, and then detached and counted. Column chart shows mean and SEM values of cell counts from four independent experiments. (C) Transfected cells were subjected to immunoblotting analysis of the anti-apoptotic protein BCL2 and the housekeeping protein β-actin. Upper panel shows representative immunoblotting images. Column chart shows mean and SEM values of BCL2 band intensity standardized against β-actin band intensity from three independent experiments. (D) Transfected cells were cultured on collagen IV coated or uncoated multi-well plates, and subjected to apoptosis assays. Images show representative staining results: nuclei of apoptotic cells are in green, and those in non-apoptotic cells are in blue. Column chart shows mean and SEM values of apoptotic cells from four independent experiments. (PDF) [file pgen.1006127.s006.pdf]

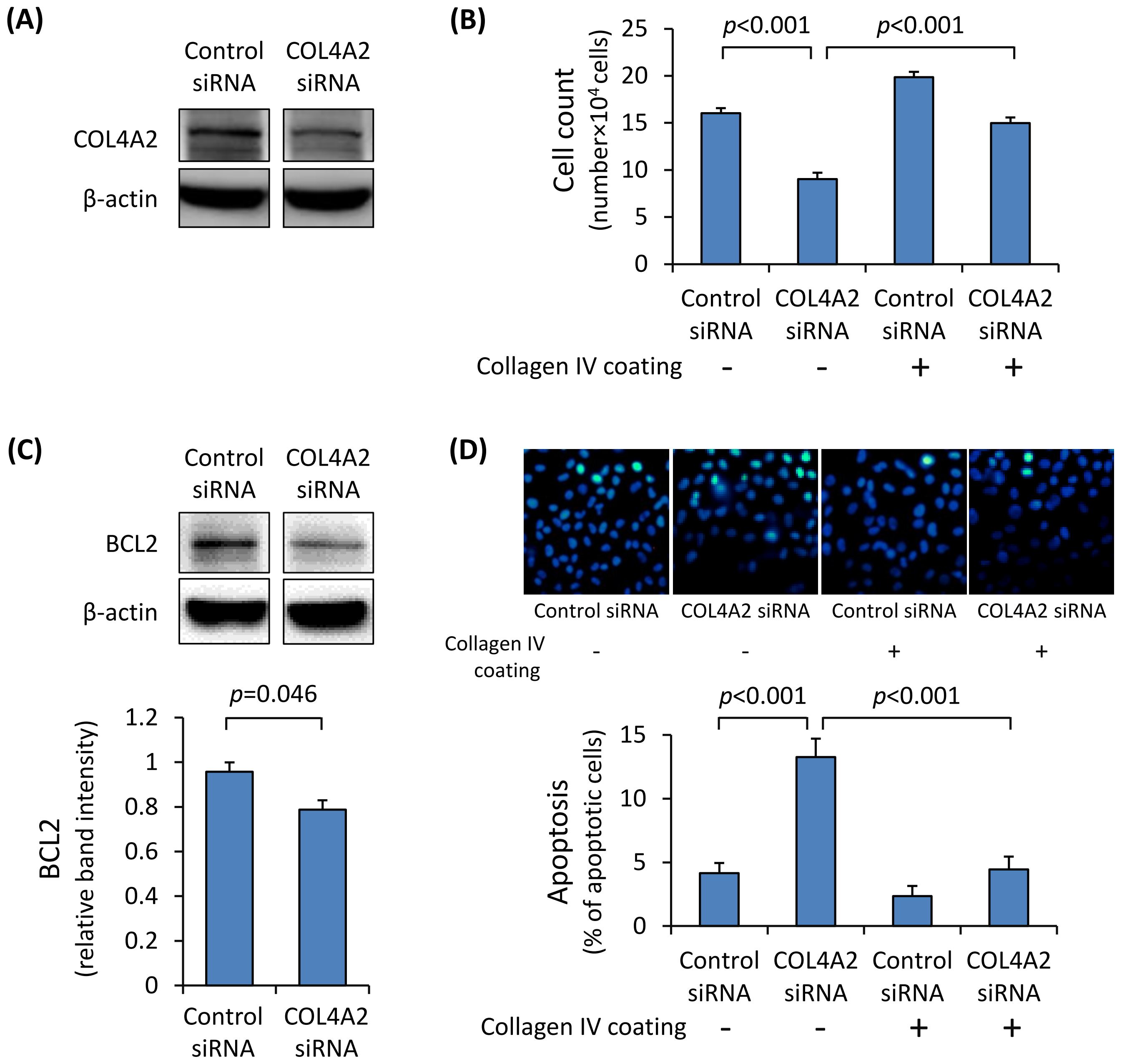

S6 Fig. Effect of COL4A2 Knockdown on Cultured Vascular SMCs.

Human aortic artery SMCs were transfected with *COL4A2* siRNA or control siRNA.

(A) Transfected cells were subjected to immunoblotting analysis of the target protein COL4A2 and the housekeeping protein β-actin. Shown in figure are representative immunoblotting images.

(B) Transfected cells (same number) were cultured on collagen IV coated or uncoated multi-well plates for 72 hours, and then detached and counted. Column chart shows mean and SEM values of cell counts from four independent experiments.

(C) Transfected cells were subjected to immunoblotting analysis of the anti-apoptotic protein BCL2 and the housekeeping protein β-actin. Upper panel shows representative immunoblotting images. Column chart shows mean and SEM values of BCL2 band intensity standardized against β-actin band intensity from three independent experiments.

(D) Transfected cells were cultured on collagen IV coated or uncoated multi-well plates, and subjected to apoptosis assays. Images show representative staining results: nuclei of apoptotic cells are in green, and those in non-apoptotic cells are in blue. Column chart shows mean and SEM values of apoptotic cells from four independent experiments.
