# Supplementary material for: Coronary-Heart-Disease-Associated Genetic Variant at the COL4A1/COL4A2 Locus Affects COL4A1/COL4A2 Expression, Vascular Cell Survival, Atherosclerotic Plaque Stability and Risk of Myocardial Infarction
Source: PLoS Genet. 2016 Jul 7;12(7):e1006127. doi: 10.1371/journal.pgen.1006127 (PMC4936713; doi:10.1371/journal.pgen.1006127)
Supplement: S11 Fig — Atherosclerotic coronary arteries from different individuals were genotyped for SNP rs4773144 and subjected to histopathological analysis. Atherosclerotic plaque cap thickness and whole intima thickness were analyzed using Image-Pro software. (A) Data from sample set one. (B) Data from sample set two. (C) Results from the two sample sets combined. Data shown are mean and SEM values in different genotype groups; p-values shown are for an additive genetic model. (PDF) [file pgen.1006127.s011.pdf]

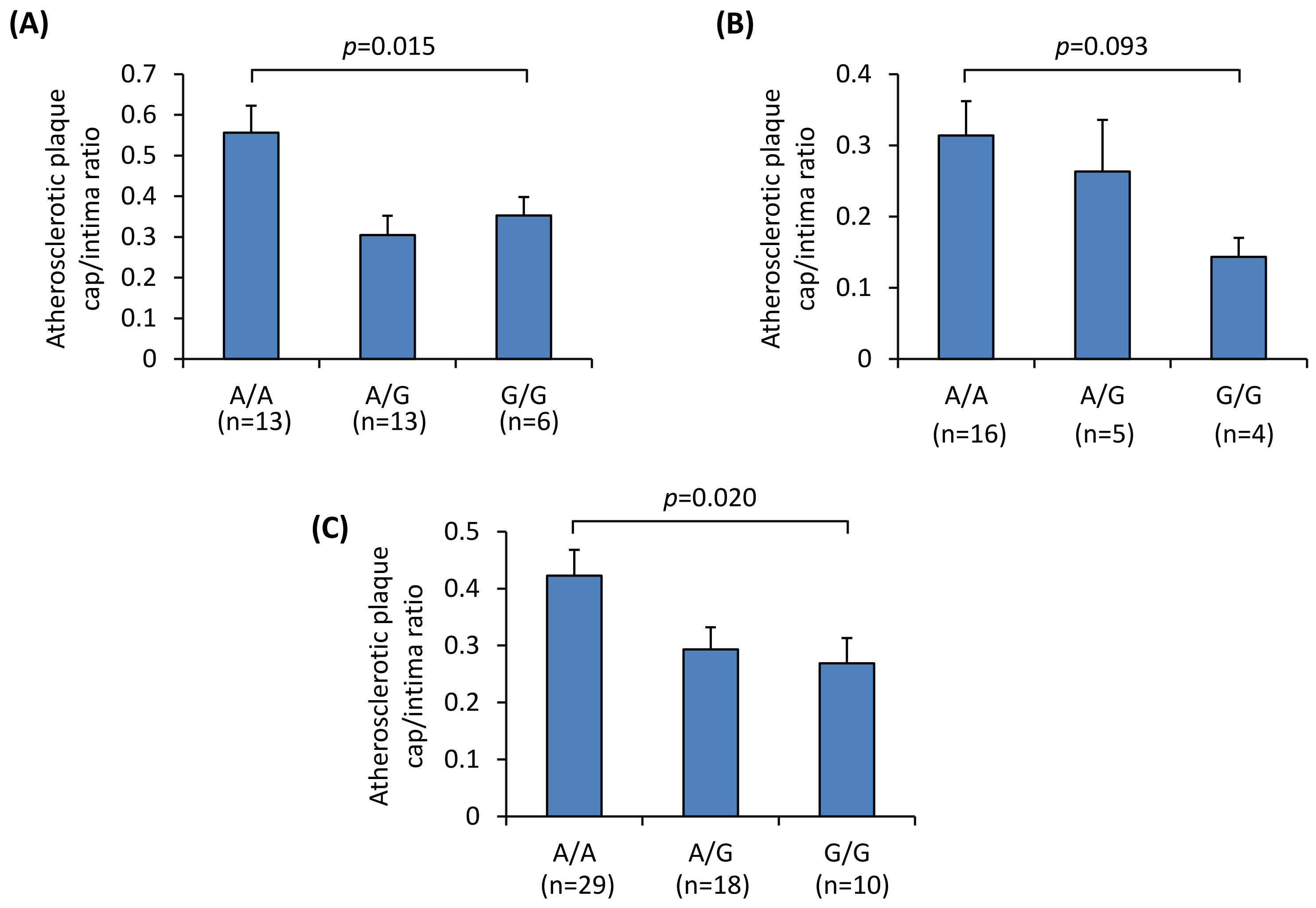

S11 Fig. Association of SNP rs4773144 with Plaque Cap/Intima Thickness Ratio.

Atherosclerotic coronary arteries from different individuals were genotyped for SNP rs4773144 and subjected to histopathological analysis. Atherosclerotic plaque cap thickness and whole intima thickness were analyzed using Image-Pro software. **(A)** Data from sample set one. **(B)** Data from sample set two. **(C)** Results from the two sample sets combined. Data shown are mean and SEM values in different genotype groups; *p*-values shown are for an additive genetic model.
